# Supplementary material for: Fermi Arcs vs. Fermi Pockets in Electron-doped Perovskite Iridates
Source: Sci Rep. 2015 Feb 23;5:8533. doi: 10.1038/srep08533 (PMC4336940; doi:10.1038/srep08533)
Supplement: Supplementary Information — for Fermi Arcs vs. Fermi Pockets in Electron-doped Perovskite Iridates [file srep08533-s1.pdf]

Supplementary Information for Fermi Arcs vs. Fermi Pockets in Electron-doped Perovskite Iridates

Junfeng He[1], H. Hafiz[2], Thomas R. Mion[1], T. Hogan[1], C. Dhital[1,6], X. Chen[1], Qisen Lin[1], M. Hashimoto[3], D. H. Lu[3], Y. Zhang[4], R. S. Markiewicz[2], A. Bansil[2], S. D. Wilson[1,5], and Rui-Hua He[1]

[1]*Department of Physics, Boston College, Chestnut Hill, MA 02467, USA*

[2]*Physics Department, Northeastern University, Boston, MA 02115, USA*

[3]*Stanford Synchrotron Radiation Lightsource, SLAC National Accelerator Laboratory, Menlo Park, CA 94025, USA*

[4]*International Center for Quantum Materials, Peking University, Beijing 100871, China*

[5]*Materials Department, University of California, Santa Barbara, CA 93106, USA*

[6]*Chemical and Engineering Materials Divison, Oak Ridge National Laboratory, Oak Ridge, TN 37831, USA*

ARPES spectra along the (0,0)-(π,π) cut at other photon energies and polarizations.

Measurements along the (0,0)-(π,π) cut (the same momentum cut as that shown in Fig. 2 of the main text) with p-polarized 23 eV and 30 eV light are shown in Fig. S1. It is clear that #1 and #2 bands are strongly suppressed with p-polarized 23 eV light (panels a and b of Fig. S1), while #3 and #4 bands are enhanced. On the other hand, the corresponding spectra at 30 eV in panels c and d of Fig. S1 preferentially enhance #1 and #2 bands. These results further highlight strong ARPES matrix element effects in this system.

Interplay of effects of structural distortion and antiferromagnetic correlations on the low-lying band structure.

Here we consider results of first-principles calculation without the presence of octahedral rotations (undistorted lattice). By considering antiferromagnetic correlations (AFM), Fermi pockets are seen to be clearly reproduced near M in panel a of Fig. S2. This is also seen in the related dispersion in panel b in which two electron-like bands appear near M, which produce the Fermi pockets. We have also carried out calculations in the paramagnetic state (PM) for the distorted lattice. Using a larger spin-orbit coupling strength, two similar electron-like bands and Fermi pockets can be produced near M (Fig. S2c). Note, however, that the calculations in panels a-c assume a fairly large value of spin-orbit coupling strength or U, and yield gap sizes and valence-band dispersions which differ substantially from the corresponding experimental results. On the other hand, if we include both a structural distortion and antiferromagnetic correlations in the calculation simultaneously, the values of U or spin-orbit coupling strength required to open a partial gap and produce Fermi pockets of a similar size are smaller [compare panel (d) with panels (b) and (c)], yielding a reasonable overall agreement with experiment as seen by comparing panel (d) here with main Fig. 3f. We thus conclude that Fermi pockets in $(Sr_{1-x}La_x)_3Ir_2O_7$ arise from a subtle interplay between structural distortion, antiferromagnetism, spin-orbit coupling, and electron correlation effects.

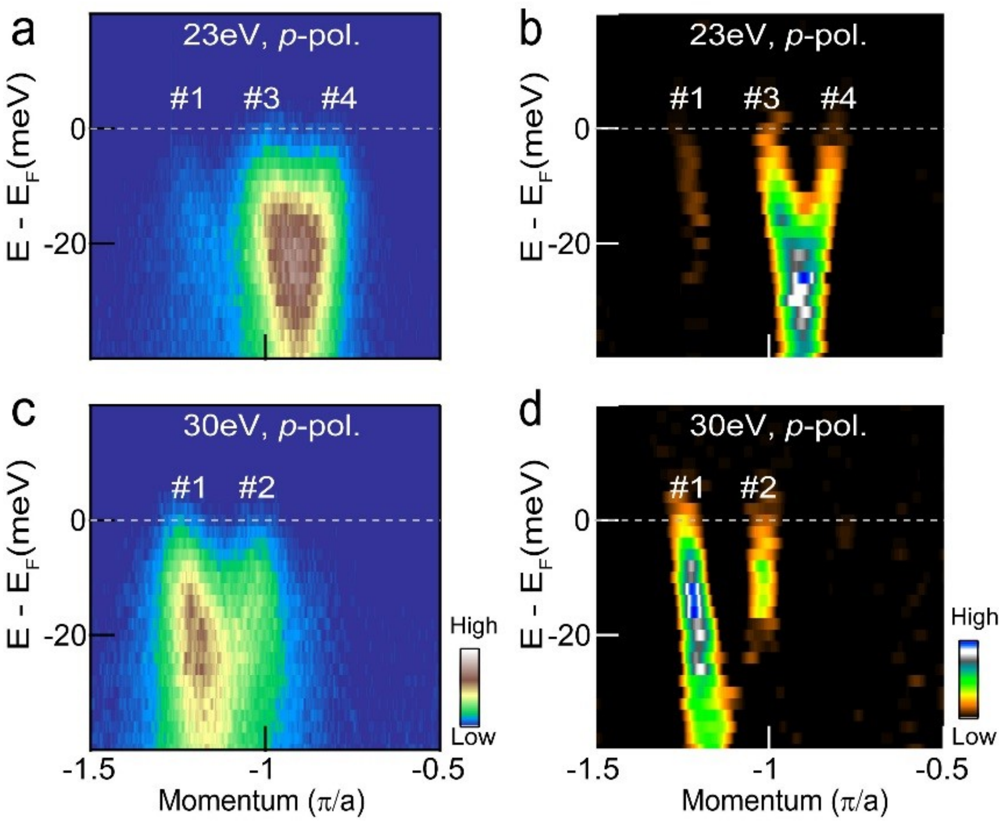

Fig.S 1: **Measurements along the (0,0)-(π,π) cut using other photon energies and polarizations.** Photoemission intensity plot (a) and the corresponding second-derivative MDC image (b) using p-polarized 23 eV light. (c & d), the same as (a & b) but with p-polarized 30 eV light. The momentum cut is the same as that shown in Fig. 2 of the main text.

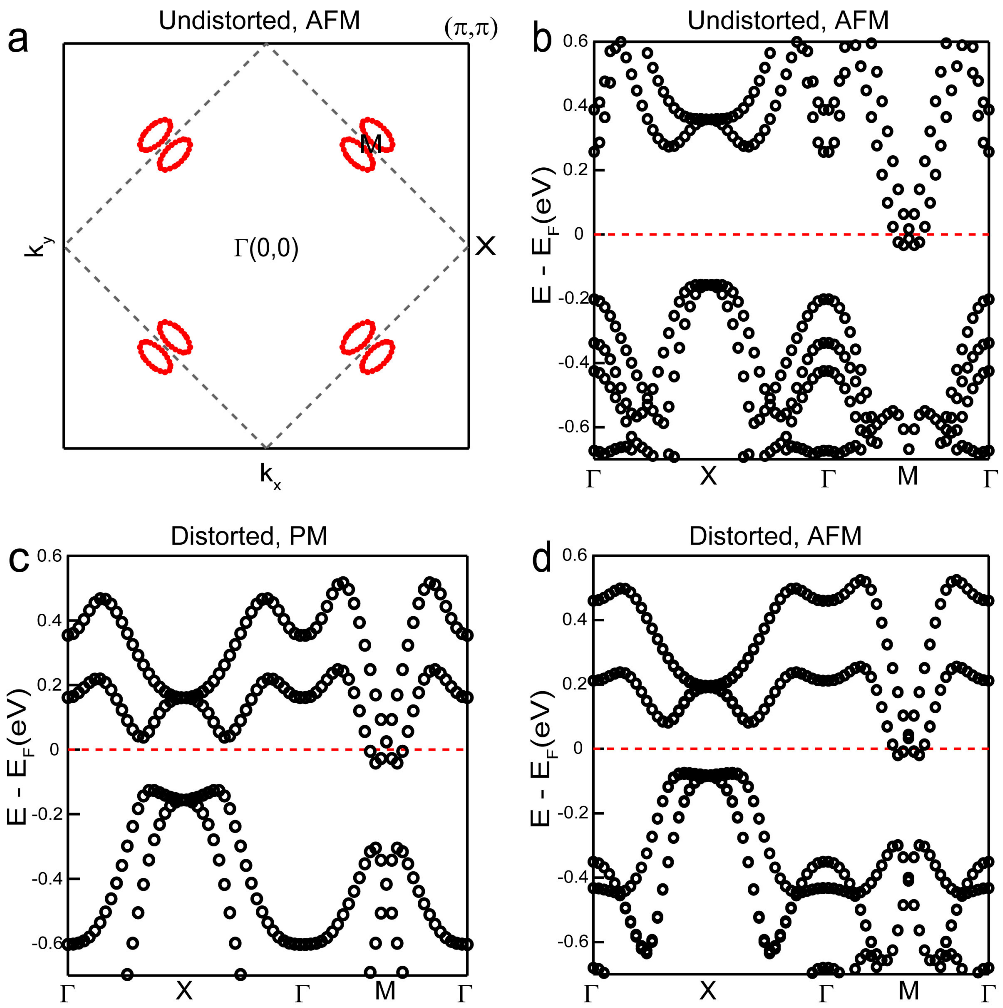

Fig.S 2: **Interplay of effects of structural distortion and antiferromagnetic correlations on the low-lying band structure.** Fermi surface (a) and dispersion along high-symmetry directions in the Brillouin zone (b) for the undistorted crystal structure (i.e., without octahedral rotations) including antiferromagnetic correlations. $U = 2$ eV and spin-orbit coupling strength is set at 1.9 times the GGA+U self-consistently obtained value, i.e. SOC=1.9. (c) Band structure for the paramagnetic state (PM) with distorted lattice. SOC=2.5. (d) Band structure when both the structural distortion and antiferromagnetic correlations ($U = 0.727$ eV, SOC=1.9) are included in the computation.
